# Development & automation of a novel [^18^F]F prosthetic group, 2-[^18^F]-fluoro-3-pyridinecarboxaldehyde, and its application to an amino(oxy)-functionalised Aβ peptide

**DOI:** 10.1016/j.apradiso.2016.07.023

**Published:** 2016-10

**Authors:** Olivia Morris, J. Gregory, M. Kadirvel, Fiona Henderson, A. Blykers, Adam McMahon, Mark Taylor, David Allsop, Stuart Allan, J. Grigg, Herve Boutin, Christian Prenant

**Affiliations:** a Wolfson Molecular Imaging Centre, CRUK/EPSRC Imaging Centre of Cambridge & Manchester, The University of Manchester, UK; b In-Vivo Cellular and Molecular Imaging Lab, Vrije Universiteit Brussel, Belgium; c Division of Biomedical and Life Sciences, The University of Lancaster, UK; d The University of Manchester, UK; e GE Healthcare, Life Sciences, Imaging R&D, The Grove Centre, Amersham, Bucks, UK

**Keywords:** [^18^F]F prosthetic group, PET, Aβ peptide, Chemical ligation, Oxime bond

## Abstract

2-[^18^F]-Fluoro-3-pyridinecarboxaldehyde ([^18^F]FPCA) is a novel, water-soluble prosthetic group. It's radiochemistry has been developed and fully-automated for application in chemoselective radiolabelling of amino(oxy)-derivatised RI-OR2-TAT peptide, (Aoa-k)-RI-OR2-TAT, using a GE TRACERlab FX-FN. RI-OR2-TAT is a brain-penetrant, retro-inverso peptide that binds to amyloid species associated with Alzheimer's Disease. Radiolabelled (Aoa-k)-RI-OR2-TAT was reproducibly synthesised and the product of the reaction with FPCA has been fully characterised. *In-vivo* biodistribution of [^18^F]RI-OR2-TAT has been measured in Wistar rats.

## 1 Background

Positron emission tomography (PET) is a highly-sensitive and quantitative molecular imaging modality, enabling non-invasive investigation of physiological events at the molecular level. The advent of novel radiotracers for use in PET has facilitated the discovery of influential pathological biomarkers. Automation of radiosynthesis aids translation from pre-clinical to clinical studies by enhancing reproducibility and reliability. Automation is, therefore, an important step in radiotracer development. The GE TRACERlab FX-FN is a popular radiosynthesis platform which is easily adaptable to automate a variety of radiosynthetic routes.

Taylor et al., 2010, Parthsarathy et al., 2013 have reported the development of RI-OR2 (Ac-rGffvlkGr-NH_2_), a retro-inverso peptide comprised of D-amino acids which confers high proteolytic stability to the peptide. RI-OR2 binds to amyloid species associated with Alzheimer's Disease (AD) with nanomolar affinity (k_d_=58–125 nM) and has been identified as an inhibitor of early-stage amyloid aggregates (Parthsarathy et al., 2013). More recently, RI-OR2 was functionalised with a retro-inverso ‘TAT’ motif to aid its crossing the blood brain barrier (BBB), resulting in the sequence Ac-rGffvlkGrrrrqrrkkrGy-NH_2,_ and a molecular weight of 2860 Da (Parthsarathy et al., 2013). ‘TAT’ is the HIV protein transduction domain and has been reported to enhance cell-permeability and aid transport across the BBB (Rizzuti et al., 2015). Radiolabelling of RI-OR2-TAT would establish its usefulness as a PET radiotracer for use in AD. In order to do this, it was first necessary to devise a radiosynthetic strategy to radiolabel RI-OR2 with the [^18^F]fluorine, a PET isotope with a appropriate radionuclide half-life (t_1/2_ 109.8 min).

**Fig. 1:**
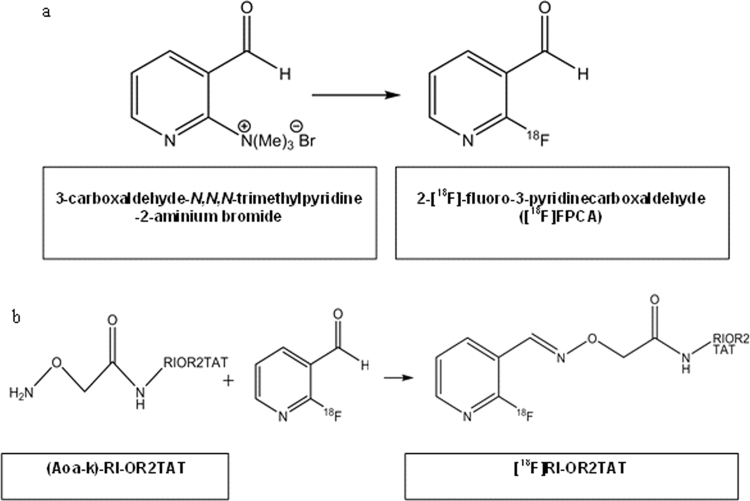
**a** Reaction pathway showing the radiosynthesis of [^18^F]FPCA **1b** Reaction pathway showing the conjugation of [^18^F]FPCA to *N*-ε-aminooxyacetyl-D-lysine (Aoa-k) modified peptide ((Aoa-k)-RI-OR2-TAT) through oxime bond formation.

Here we report a novel, water-soluble prosthetic group, 2-[^18^F]fluoro-3-pyridinecarboxaldehyde ([^18^F]FPCA) which has been developed for use in peptide and protein radiolabelling. Its solubility in aqueous media makes it highly suitable for radiolabelling peptides and proteins, such as RI-OR2-TAT, that are sensitive to organic solvent. The half-life of ^18^F (t_1/2_ 109.8 min) not only permits multi-step syntheses but is also commensurate with the biological half-life of peptides and many small proteins, such as RI-OR2-TAT. [^18^F]FPCA was produced in a one-pot, one-step direct [^18^F]fluorination of a trimethylammonium precursor, 3-carboxaldehyde-*N,N,N*-trimethylpyridine-2-aminium bromide, that was synthesised in-house. The ^18^F-fluorination of the precursor is depicted in Fig. 1a.

In order to preserve the biological activity of the peptide, it was necessary to site-specifically radiolabel RI-OR2-TAT. Therefore, the peptide was further modified with an amino(oxy)-moiety to permit chemoselective labelling with [^18^F]FPCA via oxime bond formation (Namavari et al., 2008, Poethko et al., 2004). For this, an additional D-lysine residue was linked to the C-terminal of the RI-OR2-TAT sequence, to which the amino(oxy)-functionality was added to the ε-amine group, resulting in (Aoa-k)-RI-OR2-TAT. The radiolabelling pathway can be seen in Fig. 1b. It has been reported that derivatisation of the C-terminal belonging to the ‘TAT’ fragment does not impact its ability to mediate cellular uptake (Nori et al., 2003).

Oximation of amino(oxy)-functionalised peptides with the aldehydic prosthetic group [^18^F]fluorobenzaldehyde ([^18^F]FBA) has, to date, been widely reported upon (Flavell et al., 2008, Glaser et al., 2013, Rosik et al., 2014). However, the lipophilicity of [^18^F]FBA makes radiolabeling of proteins sensitive to organic solvents both problematic and unreliable. This prompted the development of [^18^F]FPCA, on account of its enhanced hydrophilicity. Diversely functionalised ^18^F-fluoropyridine-based prosthetic groups have been used for chemoselective ^18^F-labelling of macromolecules. This includes [^18^F]FPyMe, a maleimide derivative for terminal cysteine conjugation, (de Bruin et al., 2005) and [^18^F]FPyKYNE, employed in azide-alkyne cycloaddition click chemistry (Kuhnast et al., 2008). Pyridine-based ^18^F-prosthetic groups are not only interesting in terms of the hydrophilic character but also the electronic properties of the ring which enables facile ^18^F-aromatic nucleophilic substitution.

This peptide radiolabelling strategy presented here, can be applied to both amino(oxy)-derivatised peptides, as has been described, as well as non-functionalised, native macromolecules via reductive alkylation of the *N*-terminal amine or ε-amine group of lysine residues (Means and Feeney, 1995, Prenant et al., 2008).

Here, we describe development of [^18^F]FPCA peptide radiolabelling with application to an (Aoa-k)-RI-OR2-TAT, process automation using a customised GE TRACERlab FX-FN together with *in-vitro* investigation and pre-clinical metabolite and biodistribution analysis in Wistar rats.

## 2 Methods

All solvents were purchased from Sigma-Aldrich and used without further purification. The amino(oxy)-peptide was purchased from Biomatik (Ontario, Canada) in acetate salt at a purity of >98%. [^18^F]Fluoride was produced onsite via the ^18^O(*p, n*)^18^F nuclear reaction by 16.4 MeV proton bombardment of enriched [^18^O]H_2_O using a GE PETtrace cyclotron (Bucks, UK). Analytical HPLC was performed using a Shimadzu (Milton Keynes, UK) Prominence system (LC-20AB solvent delivery system, SPD-20A dual wavelength absorbance detector) controlled by Laura 3 software (LabLogic, Sheffield, UK), Laura 3 software via a CBM-20A controller. HPLC eluate was measured for radioactivity using a Bioscan (Oxford, UK) Flowcount B-FC 3100 gamma detector. All pre-clinical PET scans were carried out using a Siemens (Oxford, UK) Inveon® PET-CT scanner. MALDI-MS was carried out using AXIMA performance MALDI-TOF MS (Shimadzu, UK). Mass spectra were acquired using a Waters SQD2 (Waters, UK) and ^1^H and ^13^C NMR spectra were recorded on a Bruker Avance 500 MHz spectrometer (Bruker, US) operated with TOPSPIN NMR software (version 2.0).

### 2.1 [^18^F]Potassium fluoride

Cyclotron produced [^18^F]fluoride was trapped on a Sep-Pak QMA cartridge (Oasis, Waters, UK) then eluted with K_2_CO_3_ solution (4 μmol, 0.4 ml) into a Reactor 1 containing 18-crown-6 (8 mg, 30 μmol) in acetonitrile (0.6 ml). The mixture was azeotropically dried with 3 sequential additions of acetonitrile (1.6 ml total) at temperature of 90 °C.

### 2.2 [^18^F]FPCA

To the reactor containing azeotropically dried [^18^F]fluoride a solution containing 3-carboxaldehyde-*N,N,N*-trimethylpyridine-2-aminium bromide (2 mg, 8 μmol) in DMSO (200 μl) was added and heated to 70 °C for 10 min [^18^F]FPCA was purified using AFFINIMIP^®^ (AFFINISEP, France) 2.0 ml SPE cartridges. Elution from the SPE cartridge was achieved using methanol (3 ml) followed by solvent evaporation at 60 °C, under vacuum for 6.5 min. The chemical identity of [^18^F]FPCA was determined by comparing its chromatographic properties with those of the isotopically unmodified FPCA (obtained from Sigma-Aldrich, UK).

### 2.3 [^18^F]RI-OR2-TAT

A solution of (Aoa-k)-RI-OR2-TAT (2 mg, 740 nmol), gentisic acid sodium salt (1 mg) anilinium chloride (2.6 mg) and citric acid (0.02 M, 250 μl) was added to the purified and dried [^18^F]FPCA (pH 2.7). The reaction mixture was heated to 50 °C for 20 min and purified using SE-HPLC (Superdex peptide 10/300 GL, PBS with 1% ascorbic acid, 1 ml/min, 280 nm, *t*_R_ 16.5 min) and analysed for quality control purposes using a Jupiter C4 column (5 μ 300 Å 250×10 mm, Phenomenex UK, Macclesfield, Cheshire, UK) and eluted using an acetonitrile gradient (10–85% over 25 min, 3 ml/min at 280 nm, *t*_R_ 13.5 min).

## 3 Synthesis of stable compounds

### 3.1 3-carboxaldehyde-*N,N,N*-trimethylpyridine-2-aminium bromide

The precursor was synthesised according to a procedure reported in the literature (Olberg et al., 2010, Yue et al., 2014). In short, 1.1 ml trimethylamine (1 M solution in THF, Fisher Scientific, UK) was added dropwise to a solution containing 2-bromo-3-pyridinecarboxaldehyde (1 mmol) in DMF. The reaction mixture was left under stirring at 70 °C for 72 h. The white precipitate was collected by filtration, washed with Et_2_O (50 ml) and cold DCM (10 ml) and dried under vacuum. The product 3-carboxaldehyde-*N,N,N-*trimethylpyridine-2-aminium bromide was obtained with 80% yields. ESI-MS: [M - Br]^+^=165. ^1^H NMR (500 Mz, DMSO-*d*_6_): 10.32 (s, 1H, CHO), 8.86 (d, 1H, J 3.0 Hz, Ar H-6), 8.80 (d, 1H, J 7.5 Hz, Ar H-4), 8.06 (dd, 1H, J 7.5, 4.5 Hz, Ar H-5), 3.71( s, 9H, NMe_3_).

^13^C NMR (125 Mz, DMSO-*d*_6_): 190.8 (C=O), 151.8 (Ar C-3), 150.6 (Ar C-6), 147.2 (Ar C-4), 126.6 (Ar C-5), 124.6 (Ar C-2), 54.0 (NMe_3_).

### 3.2 [^19^F]RI-OR2-TAT

To a solution of (Aoa-k)-RI-OR2-TAT (2.9 mg, 1.01 μmol) and anilinium chloride (1.3 mg, 10 μmol) in acetate buffer (500 μl, pH 4.7) was added 13 μl of a 0.1 M solution of 2-fluoro-3-pyridinecarboxaldehyde (Sigma Aldrich, UK) in ethanol. The mixture was incubated at 50 °C for 4 h before being purified by SE-HPLC (GE Superdex peptide 10/300 GL, PBS, 1 ml/min, 280 nm, *t*_R_ 16.5 min). The collected fraction was loaded onto an Oasis HLB cartridge (Waters) and the product was eluted with ethanol. Evaporation of the solvent and drying of the product in a vacuum desiccator yielded 1.9 mg of [^19^F]RI-OR2 (yield =64%). Labelling of (Aoa-k)-RI-OR2-TAT was verified using MALDI-MS using the following procedure. The sample (1 μl) was spotted onto a MALDI steel target plate, followed immediately by an equivalent volume of matrix (10 mg/ml α-cyano-4-hydroxycinnamic acid in 50% acetonitrile). [^19^F]RI-OR2-TAT was analysed in reflectron mode.

### 3.3 [^19^F]RI-OR2-TAT Thioflavin-T assay (ThT)

Thioflavin-T (ThT) assays were carried out at Lancaster University as previously described by (Taylor et al., 2010). In short, Aβ peptides (25 μM) were incubated with ThT (15 μM) at 25 °C in 96-well clear-bottom microtiter plates (NUNC). Inhibitor (RI-OR2-TAT or [^19^F]RI-OR2-TAT) was added in molar ratios of 1:1, 1:2, 1:10 relative to Aβ. Plates were read every 10 min (λ_ex_ 442 nm and λ_em_ 483 nm) using a BioTek Synergy plate reader (Swindon, UK) for a total of 48 h.

### 3.4 Pre-clinical PET analyses

All animal handling was in accordance with UK legislation under the 1986 Animals (Scientific Procedures) Act.

### 3.5 Pre-clinical PET

Wistar rats (n =3) were anaesthetised using isoflurane (induction 4% and maintained 1.5%) in 70% N_2_O and 30% O_2_ mixture. 44–55 MBq of [^18^F]RI-OR2-TAT was injected in the tail vein. All scans were carried out using a Siemens Inveon® PET-CT scanner. The acquisition protocol parameters consisted of a preliminary CT scan to attain attenuation correction factors followed by a 1 h PET acquisition with time coincidence window of 3.432 ns and levels of energy discrimination set between 350 keV and 650 keV. Images were reconstructed and analysed as described elsewhere (Boutin et al., 2015).

## 4 Metabolite analysis

For metabolite analysis, Sprague-Dawley rats (n=4) were used and analysis carried out as described by Cawthorne et al. (Cawthorne et al., 2011). In short, brain, liver and blood samples were taken 5 (n =2) and 20 min (n =2) post injection of [^18^F]RI-OR2-TAT. Liver and brain were quickly removed and homogenised (Ultra-turrax®, Sigma-Aldrich) in 2 ml of either ice-cold acetonitrile or PBS. After centrifugation (3 min, 9000 g, 4 °C, Thermo ALC multispeed refrigerated centrifuge PK121R (Thermo Fisher Scientific, UK)), the supernatant was separated from the pellet. For each homogenate and supernatant, an aliquot was counted using a γ-counter. Blood samples were centrifuged using a Thermo ALC multispeed refrigerated centrifuge (2200 g, 5 min) and the plasma was collected and added to either PBS or acetonitrile. Brain, liver and plasma samples in both acetonitrile and PBS were then analysed using RP-HPLC to determine the non-polar and polar metabolites respectively (Phenomenex Jupiter C4, 5 μ 300 Å 250×10 mm, acetonitrile gradient from 10 to 85% over 25 min).

## 5 Results and discussion

### 5.1 Radiochemistry development & automation

**Fig. 2:**
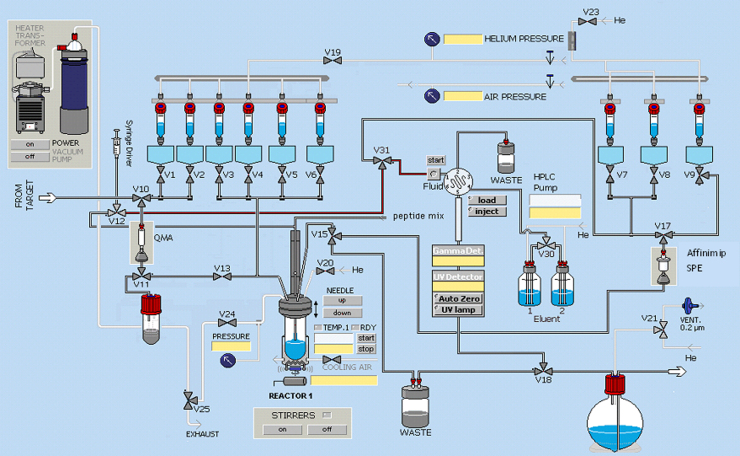
Schematic of customised GE TRACERlab FX-FN configuration permitting [^18^F]FPCA radiosynthesis, purification and isolation followed by (Aoa-k)-RI-OR2-TAT peptide radiolabelling and purification.

Fig. 2 shows the customised configuration of the GE TRACERlab FX-FN permitting [^18^F]FPCA radiosynthesis, subsequent peptide labelling and product purification.

**Fig. 3:**
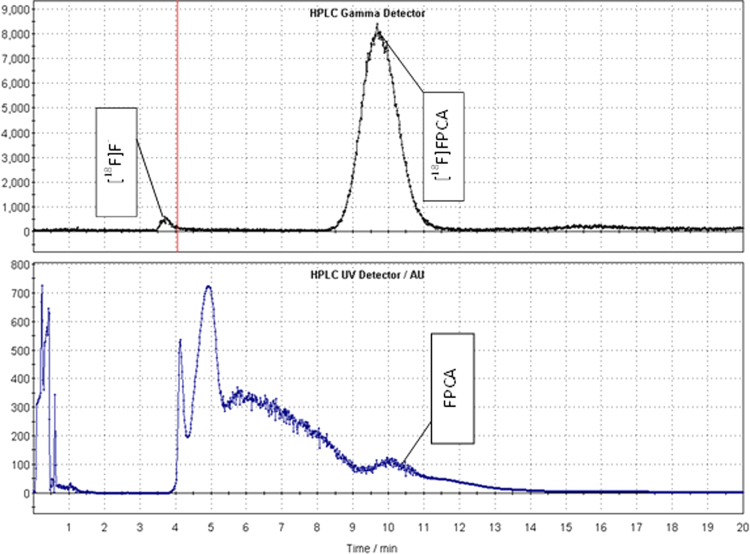
TRACERlab FX-FN radio-chromatogram trace showing crude [^18^F]fluorination mixture of [^18^F]FPCA (220 nm). The top and bottom spectra show the gamma (counts) and UV detector (AU) traces.

In initial productions [^18^F]FPCA was purified by means of RP-HPLC, followed by SPE concentration, Fig. 3 shows the chromatogram of the crude fluorination reaction mixture. According to HPLC data seen in Fig. 3, the incorporation of [^18^F]fluoride is higher than 85%, (n=20) and remained reproducible. [^18^F]FPCA can be seen with a retention time of 9.5 min with residual [^18^F]fluoride seen at 3.5 min

Replacement of RP-HPLC purification with a SPE method was implemented in the interest of time reduction but also to simplify automation and improve reproducibility. Due to the polar nature of the prosthetic group, it was not suitable for use with RP-SPE cartridges and use of a hydrophobic-lipophilic balance (HLB) cartridge resulted in unwanted water content in the eluted fraction of [^18^F]FPCA. Subsequent evaporation of elution solvent proved very difficult and problematic for automation, owing to varying volumes between productions. The variable volumes of co-eluted water meant that evaporation times and aqueous methanol volumes were inconsistent between reactions. As a result, a vigorous drying step was required which caused a significant loss in radioactivity and caused a marked reduction in yield. Under other conditions, excess water remaining after incomplete evaporation acted to dilute the peptide labelling reaction mixture and, again, resulted in lower radiochemical yields.

For this reason, the AFFINIMIP® SPE ^18^F-Aromatic Nucleophilic Substitution 2.0 ml cartridge was assessed and found to achieve both good retention and resolution of [^18^F]FPCA from the trimethylammonium precursor. AFFINIMIP® SPE ^18^F-Aromatic Nucleophilic Substitution 2.0 ml cartridges comprise a molecular imprinted polymer that has been validated for use with the radiotracers [^18^F]fluorobenzaldehyde (FBA) and [^18^F]ethyl-4-fluorobenzoate. The prosthetic groups are produced from an aromatic nucleophilic substitution with the corresponding ammonium-based precursor. Good separation of the ^18^F-prosthetic groups, from the ammonium precursor as well as the phenol- and dialkylamino-based impurities, is achieved using the cartridge. The radiochemical purity (RCP) and chemical purity of SPE-purified [^18^F]FPCA was verified using RP-HPLC (data not shown). Reactor 1 was cleaned by addition of ethanol (2 ml) from Vial 5 before re-addition of [^18^F]FPCA from the SPE cartridge for subsequent conjugation to (Aoa-k)-RI-OR2-TAT. This ensured that impurities from the fluorination of FPCA, that could impair peptide radiolabelling, were minimised.

An AFFINIMIP® SPE ^18^F-Aromatic Nucleophilic Substitution 0.7 ml cartridge was additionally assessed in the application of [^18^F]FPCA purification. It was found that elution from the 0.7 ml cartridge was more efficient and RCY of [^18^F]FPCA increased to 43%, however resolution from the precursor was poor which rendered the cartridge unsuitable in this application. The loss in RCY of [^18^F]FPCA using a 2.0 ml AFFINIMIP® cartridge was preferable owing to the high chemical purity that was achieved.

Despite high yields of [^18^F]FPCA, as observed in Fig. 3, RCY of 28±2%, (decay corrected, n=10) were obtained attributable to loss during SPE purification and solvent evaporation steps. However, the AFFINIMIP® SPE ^18^F-Aromatic Nucleophilic Substitution cartridge has not been produced specifically for in FPCA purification resulting in low recovery of [^18^F]FPCA. This would be markedly improved by using a bespoke FPCA specific molecular imprinted polymer cartridge.

Despite the loss of [^18^F]FPCA owing to purification and solvent evaporation, [^18^F]FPCA presents as an alternative water-soluble [^18^F]F prosthetic group for use with peptides and proteins that are sensitive to organic solvents.

**Fig. 4:**
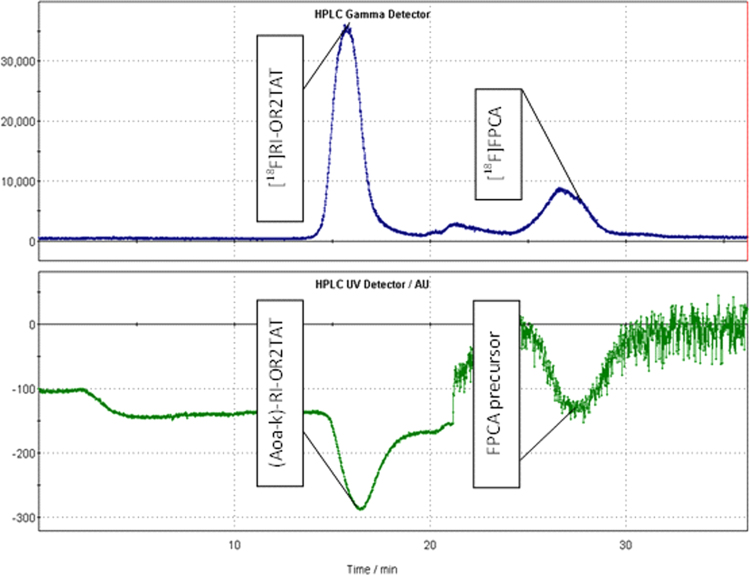
TRACERlab FX-FN radio-chromatogram trace showing SE-HPLC radio-chromatogram of [^18^F]RI-OR2-TAT.

Fig. 4 shows the SE-HPLC radio-chromatogram of the crude [^18^F]RI-OR2-TAT radiolabelling mixture. SE-HPLC was used to both purify and formulate the final radiolabelled peptide product through use of PBS eluent. The radiolabelling efficiency of the peptide with [^18^F]FPCA achieved 22.5±3% and, based on 5 syntheses, was reproducible. [^18^F]RI-OR2-TAT and [^18^F]FPCA can be seen with a retention times of 16.5 min and at 27 min respectively. A negative UV peak is observed for both the unlabelled (Aoa-k)-RI-OR2-TAT and FPCA precursor attributable to the high absorbance density of the ascorbate-containing mobile phase. RP-SPE cartridges were also assessed for their suitability in purifying [^18^F]RI-OR2-TAT. The radiolabelled peptide was strongly retained and required a large volume of organic eluent, additionally co-elution of more lipophilic components and unreacted [^18^F]FPCA was observed resulting in sub-standard radiochemical purity. Furthermore, [^18^F]FPCA radiosynthesis was developed and automated with a view to radiolabelling further peptides and proteins, in this regard SE-HPLC serves as a method by which larger or organic-sensitive proteins can be purified and formulated.

RP-HPLC was used to assess the RCP of the final formulated [^18^F]RI-OR2-TAT product for quality control purposes. The UV-radiochromatogram can be seen in the Supplementary Material (Figure. a). The Figure shows a large initial peak attributable to ascorbate and two peaks at 12.75 and 13.5 min, identified as (Aoa-k)-RI-OR2-TAT and [^18/19^F]RI-OR2-TAT respectively with a RCP >90%.

The overall [^18^F]RI-OR2-TAT RCY achieved using the automated platform was 12±2% within 2 h from end of bombardment, starting from 25 to 30 GBq of [^18^F]fluoride (decay corrected, n=5). The specific activity of [^18^F]RI-OR2-TAT was calculated to be 3.2±1.3 GBq/μmol (at end of synthesis).

Despite the appreciable radiolabelling efficiencies of [^18^F]FPCA and [^18^F]RI-OR2-TAT, the overall yield using the fully-automated platform was lower than expected and largely attributable to suboptimal elution of [^18^F]FPCA from the AFFINIMIP® SPE cartridge. Overall radiolabelling yields using a semi-automated synthesis, used during early developmental stages, achieved 20±5% (decay corrected, n=5) starting with 100 MBq of [^18^F]FPCA. In which, [^18^F]FPCA synthesis and SPE purification was achieved using the automated platform and [^18^F]FPCA evaporation and peptide radiolabelling was completed manually (data not shown).

**Fig. 5:**
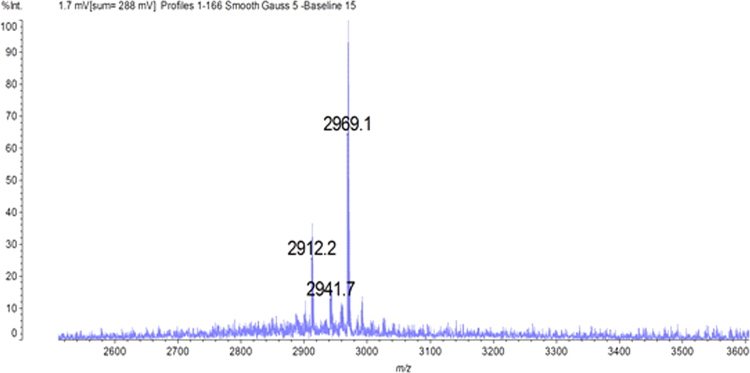
MALDI-MS analysis of (Aoa-k)-RI-OR2-TAT peptide labelled with isotopically unchanged FPCA ([^19^F]FPCA). [^19^F]RI-OR2-TAT is shown at 2969 m/z (M+H)^+^.

Fig. 5 shows the MALDI-MS analysis of [^19^F]RI-OR2-TAT peptide. The ^19^F-labelled peptide can be seen at 2969 m/z (M+H)^+^. The results of the MALDI-MS confirm labelling of (Aoa-k)-RI-OR2-TAT with FPCA.

### 5.2 Application of radiochemistry to RI-OR2-TAT

#### 5.2.1 ThT assay

A ThT assay was used in the original publications to assess the potency of RI-OR2 and RI-OR2-TAT against amyloid (Aβ) plaque aggregation (Parthsarathy et al., 2013, Taylor et al., 2010). ThT has affinity for amyloid plaque, but not for unaggregated amyloid species, therefore the fluorescent intensity of a ThT corresponds to the degree of amyloid aggregation. Although the ThT assay does not provide evidence for specific *vs* non-specific binding of the RI-OR2-TAT analogues, it does confirm their reactivity towards amyloid species and permits a direct comparison of RI-OR2-TAT and [^19^F]RI-OR2-TAT. Results of the ThT assay can be used to verify that the radiolabelling method has not modified the characteristics of the peptide.

**Fig. 6:**
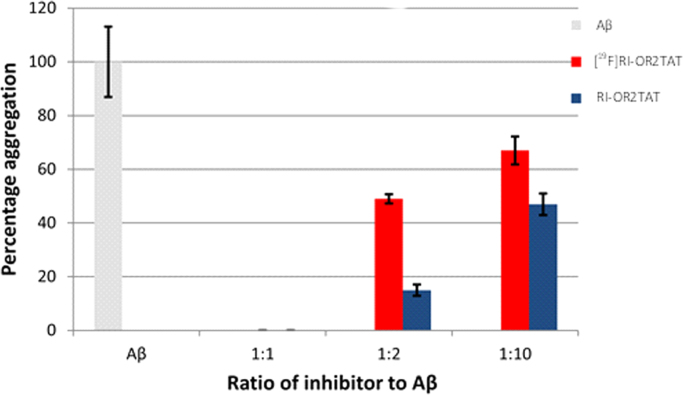
Results of the Thioflavin-T assay (ThT) showing percentage inhibition of amyloid aggregation in presence of either [^19^F]RI-OR2-TAT (red) or non-functionalised RI-OR2-TAT (blue). Amyloid aggregation in the absence of inhibitor (RI-OR2-TAT) is shown in grey. (For interpretation of the references to color in this figure legend, the reader is referred to the web version of this article.)

The results of the ThT assay can be seen in Fig. 6. The data indicate the percentage inhibition of amyloid aggregation in the presence of RI-OR2-TAT or cold labelled [^19^F]RI-OR2-TAT, shown in blue and red respectively, at different inhibitor: Aβ molar ratios. Aβ aggregation, in the absence of inhibitor, can be seen in grey. The chart shows that the inhibition efficiency of [^19^F]RI-OR2-TAT is reduced 3-fold at an inhibitor: Aβ molar ratio of 1:2. Although the ability of the modified peptide is lowered, this assay suggests [^19^F]RI-OR2-TAT still binds, demonstrating the potential of the ^18^F-analogue as a PET tracer.

#### 5.2.2 Pre-clinical results

**Fig. 7:**
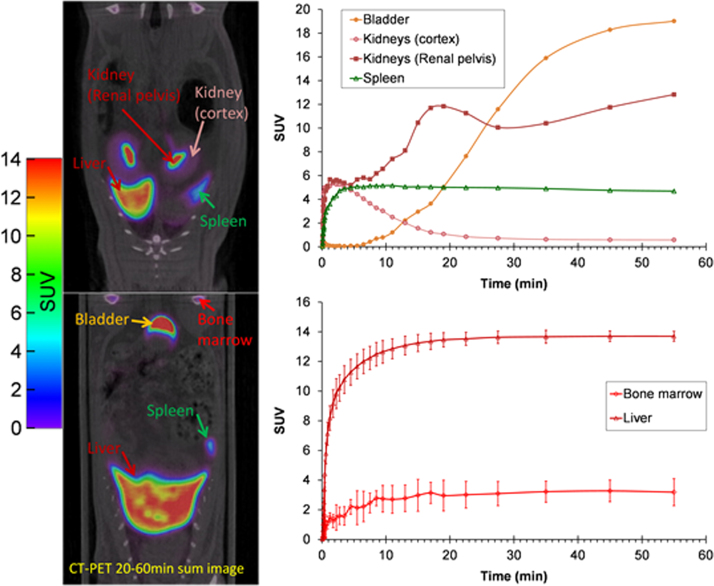
Representative sum images (20–60 min) of a Wistar rat after injection of [^18^F]RI-OR2-TAT. Time activity curves in bladder, kidneys, and spleen are from 1 animal. Data for bone marrow and liver are expressed as SUV (mean±SD of 3 rats).

**Fig. 8:**
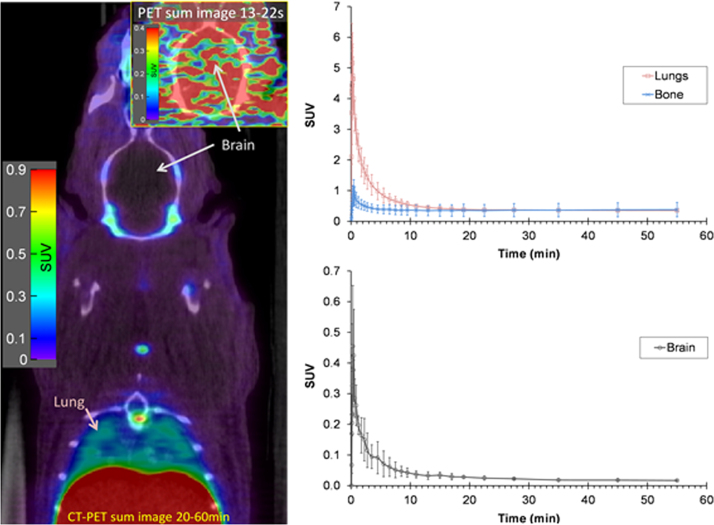
Representative sum images 20–60 min post-injection (insert: 13–22 s post-injection) of [^18^F]RI-OR2-TAT uptake in brain, bone and lungs (mean±SD, n=2 for brain, n=3 for bone and lungs).

*In-vivo* biodistribution of [^18^F]RI-OR2-TAT was performed in Wistar rats and results of which can be seen in Fig. 7 and Table 1. Overall, the highest uptake was observed in liver, with a rapid uptake reaching a plateau within 10 min post-injection, followed by bladder (urine), kidneys, spleen, bone marrow, lungs, bone and brain (Fig. 7, Fig. 8). Some uptake could be observed in bone marrow but was distinctively higher than the low uptake observed in bone (3.188±0.912 SUV and 0.380±0.237 SUV respectively 60 min post-injection) (Figs. 7D and 8B). Our measures by γ-counting of liver and brain samples were in good agreement with our PET quantification (Table 1). The nature of the retro-inverso peptide is such that hepatobiliary clearance is a main route of excretion instead of renal excretion as is often observed with small peptides. The high proteolytic stability of peptide owes to the absence of blood proteases able to recognise its D-amino acid peptide bonds. It is hypothesised that the peptide accumulates in the liver before being excreted through the intestinal tract via the bile.

Defluorination is a key metabolic process that will limit the utility of any ^18^F-labelled radiotracer and is detected when accumulation of radioactivity is observed within the bone. However Fig. 7, Fig. 8 show relatively little bone uptake thereby demonstrating stability of [^18^F]FPCA to defluorination. An evaluation of [^18^F]fluoropyridines by (Dolle, 2007) remarks upon the metabolic resistance of the ^18^F-fluoride radiolabel in the ortho-position of pyridine-based radiotracers.

Fig. 8A and C show the distribution of [^18^F]RI-OR2-TAT in rat brain. As can be seen, the brain pharmacokinetic follows a rapid peak (within 30 s post-injection) of uptake of [^18^F]RI-OR2-TAT corresponding to the first pass of the peptide with the blood flow followed by a rapid washout (Fig. 8C). After 10 min, very little brain retention of [^18^F]RI-OR2-TAT is observed. This was as expected due to absence of AD pathology in the brains of Wistar rats. The specific activity of the [^18^F]RI-OR2-TAT is currently low, however RI-OR2-TAT binds to Aβ plaques but also to Aβ oligomers. Binding to such a complex is not readily saturable, as is expected with receptor-based binding (*i.e.* there is no 1–1 stoichiometry but multiple binding sites per plaques or oligomers). Consequently, it is important to qualify that the specific activity of [^18^F]RI-OR2-TAT does not contribute to poor brain signal. Despite the presence of multiple binding sites, transportation of [^18^F]RI-OR2 across the BBB maybe hindered by competition of [^18^F]RI-OR2-TAT with unlabelled (Aoa-k)-RI-OR2-TAT. Therefore, an improvement in specific activity by separation of labelled and unlabelled peptide would safeguard against issues of radiotracer concentration affecting its pre-clinical performance.

Table 2 shows the analysis of the polar and non-polar fractions in the brain, liver and plasma samples collected in PBS and acetonitrile portions respectively at 5 and 20 min post-injection. In liver and plasma, the radioactivity detected is almost exclusively a polar species that has been identified using RP-HPLC as parent [^18^F]RI-OR2-TAT; no other radiolabelled component was identified using RP-HPLC (Supplementary Figure. b). In the brain, some non-polar species were detected in higher proportions than in the plasma or liver. This suggests the presence more lipophilic compounds which traverse the BBB more easily. Considering the extremely low level of radioactivity detected in the brain (0.017±0.002 SUV 60 min post-injection), further evaluation is needed to deduce the impact of such compounds on the signal to noise ratio in models of AD with detectable β-amyloid. The results of this analysis, however, confirm the *in-vivo* stability of both the peptide and the [^18^F]FPCA radiolabel.

## 6 Conclusion

We report the radiosynthesis of a novel, water-soluble prosthetic group, [^18^F]FPCA, and its automation using a GE TRACERlab FX-FN. [^18^F]Fluoride radiolabelling efficiency of [^18^F]FPCA achieved yields >85% (n=20). Purified and dried [^18^F]FPCA was obtained within 45 min from end of bombardment with RCP of approx. 95% and RCY of 28%±2 (decay-corrected, n=10). Overall RCYs of the [^18^F]RI-OR2-TAT achieved were 12±2% within 2 h, starting from 25 to 30 GBq of [^18^F]fluoride (decay corrected, n=5).

The pre-clinical study of [^18^F]RI-OR2-TAT demonstrates *in-vivo* stability of the tracer and primarily hepatobiliary excretion of the intact peptide owing to its retro-inverso amino-acid sequence. Very low brain penetration was observed conceivably resulting from the low specific activity. Further work is in progress to improve the specific activity, by means of separation of the labelled and unlabelled peptide, and assess the tracer in animal models of AD.

Development and automation of [^18^F]FPCA radiochemistry has produced reproducible radiolabelling of (Aoa-k)-RI-OR2-TAT. This presents [^18^F]FPCA radiochemistry and automation as a useful and generic ^18^F-prosthetic group for use in peptide and protein radiolabelling via both oxime-bond formation, as has been described in this article, or reductive alkylation.

## Competing interests

Lancaster University, David Allsop and Mark Taylor have RI-OR2-liposome patent applications (WO2013/054110, US2014/356418 and EP2766094). No other authors have any competing interests to declare.

## Figures and Tables

**Table 1:** Biodistribution by γ-counting of [^18^F]RI-OR2-TAT in brain, liver, whole-blood and plasma, and plasma to blood ratio 5 and 20 min post-injection (n=2 per time-point, data expressed as SUV, mean±SD).

	Polar	
	5 min	20 min
Brain	0.096±0.001	0.068±0.023
Liver	15.38±0.97	16.34±1.15
Whole-blood	2.262±0.572	0.189±0.080
Plasma	3.336±0.873	0.340±0.183
Plasma/blood ratio	1.47±0.01	1.74±0.23

**Table 2:** Analysis of polar (PBS) and non-polar fractions (ACN) of [^18^F]RI-OR2-TAT taken from brain, liver and plasma 5 and 20 min post-injection (n=2 per time-point, data expressed as mean±SD).

	Polar		Non-polar	
	5 min	20 min	5 min	20 min
Brain	77±3%	66±1%	23±3%	34±1%
Liver	97±2%	94±3%	3±2%	6±3%
Plasma	96±4%	76±10%	4±4%	24±10%

## Appendix A Supplementary material

Supplementary material: **Fig A** Quality control RP-HPLC radio-chromatogram of [^18^F]RI-OR2-TAT.

Supplementary material: Fig B RP-HPLC radio-chromatogram of a liver sample showing parent [^18^F]RI-OR2-TAT, suggestive of peptide stability in plasma.
