# Supplementary figures and images for: Development & automation of a novel [18F]F prosthetic group, 2-[18F]-fluoro-3-pyridinecarboxaldehyde, and its application to an amino(oxy)-functionalised Aβ peptide
Source: Appl Radiat Isot. 2016 Oct;116:120–7. doi: 10.1016/j.apradiso.2016.07.023 (PMC5034901; doi:10.1016/j.apradiso.2016.07.023)

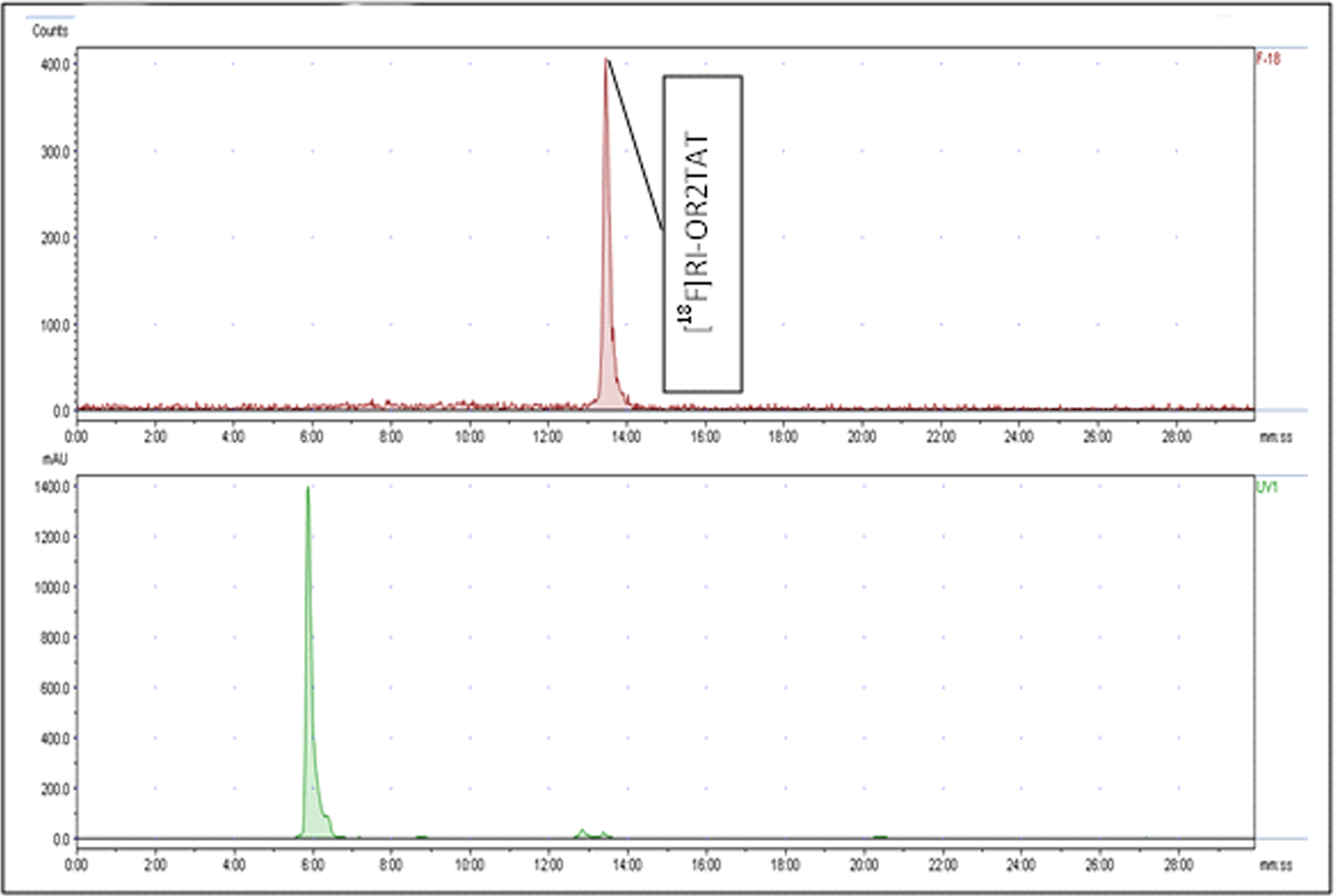

Supplement: Supplementary file 1 — Supplementary material: Fig A Quality control RP-HPLC radio-chromatogram of [18F]RI-OR2-TAT. [file mmc1.zip › mmc1.tif]

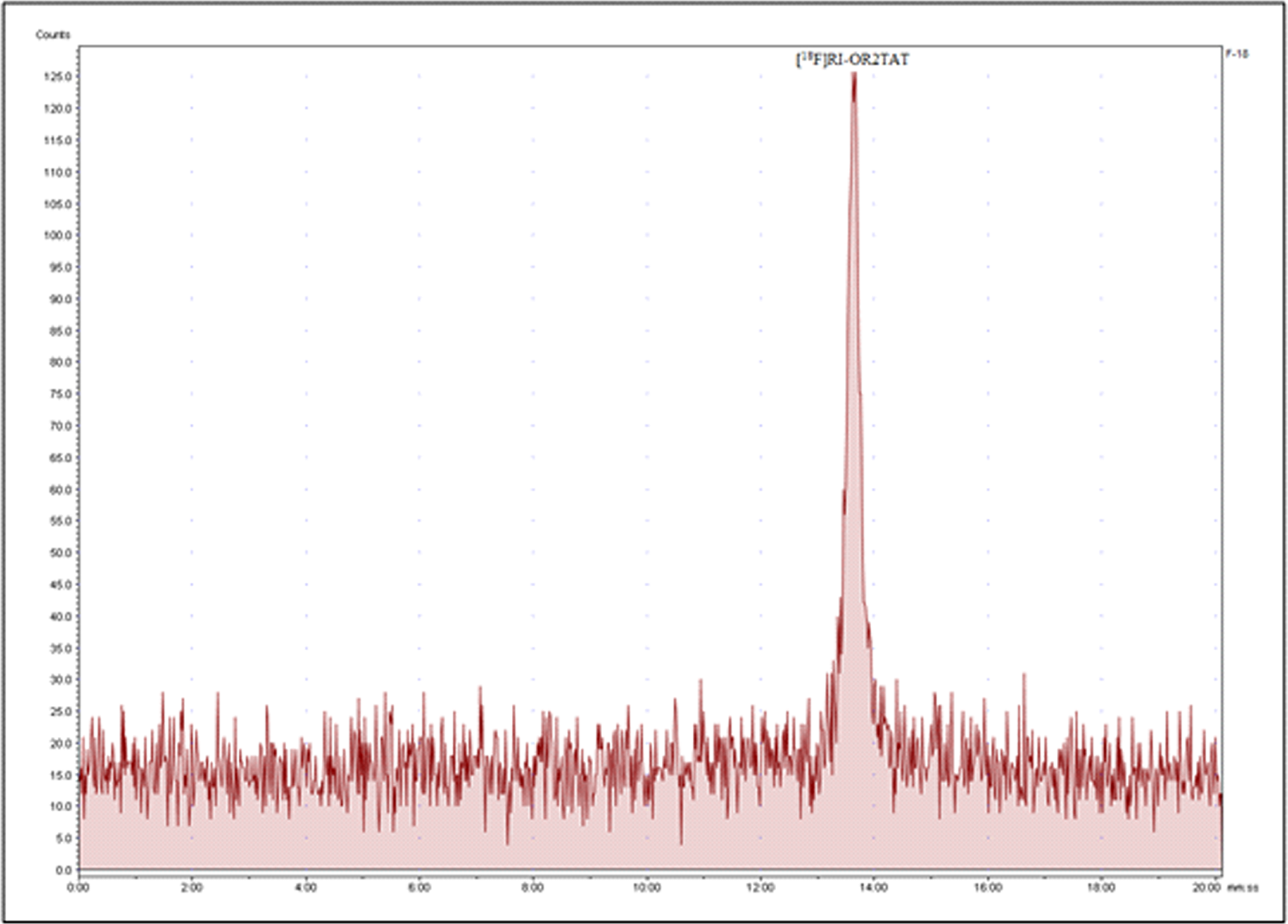

Supplement: Supplementary file 2 — Supplementary material: Fig B RP-HPLC radio-chromatogram of a liver sample showing parent [18F]RI-OR2-TAT, suggestive of peptide stability in plasma. [file mmc2.zip › mmc2.tif]
